# Supplementary material for: Retrospective Study of the Upsurge of Enterovirus D68 Clade D1 among Adults (2014–2018)
Source: Viruses. 2021 Aug 13;13(8):1607. doi: 10.3390/v13081607 (PMC8402803; doi:10.3390/v13081607)
Supplement: Supplementary file 1 [file viruses-13-01607-s001.zip › viruses-1308996-supplementary.pdf]

Supplementary materials

Retrospective study of the upsurge of enterovirus D68 clade D1 among adults (2014-2018)

Maxime Duval, Audrey Mirand, Olivier Lesens, Jacques-Olivier Bay, Denis Caillaud, Denis Gallot, Alexandre Lautrette, Sylvie Montcouquiol, Jeannot Schmidt, Carole Egron, Gwendoline Jugie, Maxime Bisseux, Christine Archimbaud, Céline Lambert, Cécile Henquell, jean-Luc Bailly

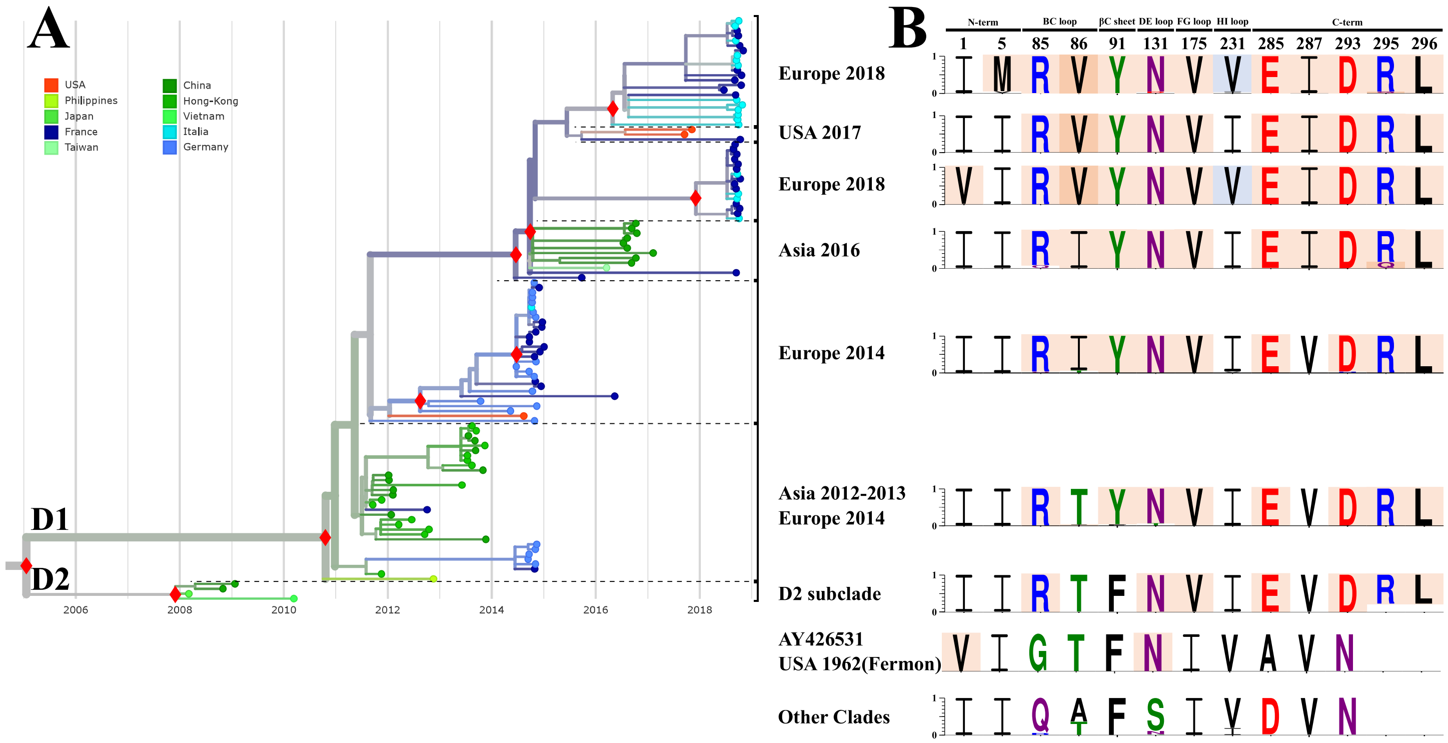

Figure S1. Phylogeny of EV-D68 clade D reconstructed from the VP1 gene molecular variation of VP1 protein.
(**A**) The phylogenetic tree was obtained with the Nextstrain pipeline [11] using a global dataset of 1433 EV-D68 whole VP1 sequences available in Genbank (as of 01.02.2020) including 64 sequences obtained in this study. The solid red diamonds indicate a node consistency, posterior probability ≥ 0.95, assessed using BEAST2 software. The branches are coloured according to the geographic origin. (**B**) The molecular analysis was performed to identify amino acid residues specific to clade D sequences. The amino acid positions related to sequence clusters are expressed in percentages on the graph with Weblogo 3 [14]. Amino acid residues with a pink background are specific to clade D and amino acids with a blue background are shared between different clades. Amino acids are coloured according to their chemistry.

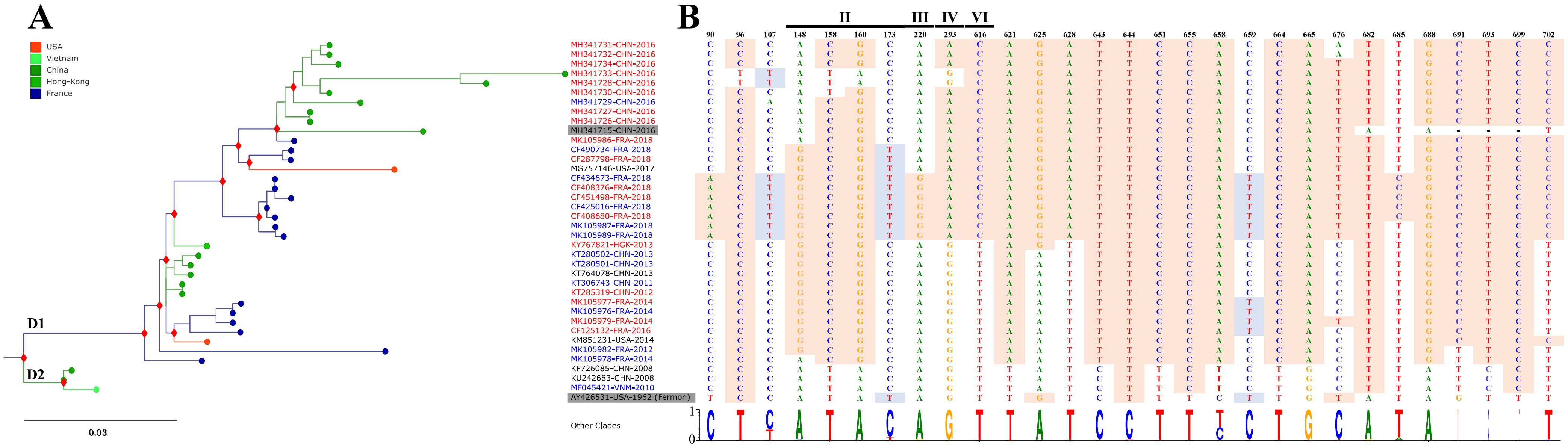

Figure S2. Phylogeny of EV-D68 clade D reconstructed from the 5' untranslated region (5'UTR) and variations in the 5'UTR sequences. (**A**) The phylogenetic tree was obtained with the program PhyML v3.0. [13] using a global dataset of 660 EV-D68 whole 5'UTR sequences available in Genbank (as of 01.02.2020) including 30 sequences obtained in this study. The solid red diamonds indicate a node consistency, posterior probability ≥ 0.94, assessed using the program PhyML v3.0 [13]. The colours of sequence names match with age groups of related patients: red and blue colours match with adult (>16 years) and children (≤16 years) patients, respectively. Sequence names in black had no corresponding information on related patient age. The branches are coloured according to the geographic origin. (**B**) The molecular analysis was performed to identify positions having specific nucleotide changes. The nucleotide positions related to sequence clusters are expressed in percentages on the graph with Weblogo 3 [14]. Nucleotides with a pink background are specific to clade D and nucleotides with a blue background are shared between different clades.

Table S1. In-house primers used for enterovirus D68 genome amplification.

Reverse transcription into DNA	Reverse	5′-TTT-TTT-TTT-TTT-TTT-TTT-TTT-TTT-TGG-YCC-CCA-AGT-GRC-CAA-AAT-TTA-CCT-C-3′	3′UTR positions 7341-7367
Near full-length genome amplification	Forward	5′-GAC-AGC-TTA-TCA-TCG-TAA-TAC-GAC-TCA-CTA-TAG-GGT-TAA-AAC-AGC-CTT-GGG-GTT-G-3′	5′UTR positions 1-20
	Reverse	5′-CGT-CTA-AGA-CTA-GAR-TAT-GCA-GGT-AGT-G-3′	3D gene positions 7250-7277
Whole genome amplification [a]	Reverse	5′-GGY-CCC-CAA-GTG-RCC-AAA-ATT-TAC-CTC-3′	3′UTR positions 7341-7367
3′ untranslated region amplification [b]	Forward	5′-ATT-AGT-AAT-GAC-ACC-AGC-3′	3D gene positions 6966-6983
	Reverse	5′-CAG-GAA-ACA-GCT-ATG-ACC-GTT-TTT-TTT-TTT-TTT-TTT-T-3′	3′UTR poly(A) tail

The genomic coordinates of primers were defined on the whole genome sequence of the Fermon prototype strain (accession number AY426531)

[a] Same forward primer used than for near full-length genome amplification.

[b] Reverse transcription into DNA was performed with the same reverse primer used for amplification and obtained from Tan, Y. et al. 2016 [32]

Table S1 : Respiratory specimens available for isolation and/or whole genome sequencing and clinical characteristics of related 46 patients.

Specimen	Collection date	Age (years)	Sex	Clinical presentations	Specimen material	Ct	Strain designation	Subclade	Accession number
1	05/10/2014	5·74	F	URTI, asthma, respiratory distress	NPA	NA	CF279027	B2	LN681325
2	14/10/2014	4·43	M	URTI, asthma, respiratory distress	NPA	NA	CF287062	B1	LN681327
3	25/10/2014	0·10	F	URTI, fever	NPA	NA	CF298032	B1	LN681332
4	02/11/2014	7·83	F	Asthma, respiratory distress	NPA	NA	CF307209	B2	MT791927
5	04/05/2016	53·60	F	COPD exacerbation, respiratory distress	NPS	20·87	CF125132	D1	MT791934
6	01/07/2016	0·17	M	URTI, bronchitis-bronchiolitis, fever	NPA	20·21	CF183054	B3	MT791933
7	06/07/2016	55·96	M	Pneumopathy, respiratory distress, fever	NPS	NA	CF188029	B3	MT795864
8	07/07/2016	1·92	M	Asthma, respiratory distress, fever	NPA	NA	CF190038	B3	MT791932
9	09/07/2016	4·00	M	URTI, asthma, respiratory distress, fever	NPA	22·62	CF193002	B3	MT789734
10	11/07/2016	78·00	F	Pneumopathy, cerebilitis, altered mental status, fever	BAF	NA	CF193158	B3	MT789735
11	11/07/2016	1·42	F	Asthma	NPA	NA	CF194006	B3	MT791930
12	12/07/2016	1·92	M	URTI, asthma, fever	NPS	24·51	CF195004	B3	MT789737
13	14/07/2016	6·21	F	URTI, asthma, fever	NPA	21·46	CF197014	B3	MT795863
14	13/08/2016	0·04	F	Bronchitis-bronchiolitis, fever	NPA	16·99	CF226028	B3	MT791929
15	11/09/2016	0·10	M	URTI	NPS	21·04	CF256006	B3	NA
16	15/09/2016	3·90	M	Asthma	NPA	NA	CF259082	B3	NA
17	22/09/2016	0·10	F	URTI, digestive signs, fever	NPA	22·82	CF266116	B3	MT789736
18	25/09/2016	0·17	M	URTI, bronchitis-bronchiolitis, respiratory distress	NPA	21·13	CF270004	B3	MT791928
19	29/09/2016	50·14	F	URTI, pneumopathy, fever	NPS	32·72	CF273012	B3	MT795868
20	02/10/2016	0·14	M	URTI, bronchitis-bronchiolitis	NPA	22·89	CF277014	B3	MT795867
21	02/10/2016	0·17	F	URTI	NPA	29·84	CF277009	B3	NA
22	05/10/2016	0·42	F	URTI, respiratory distress, infant sudden death	NPA	38·69	CF279125	UT	NA
23	12/10/2016	3·58	M	URTI, asthma, fever	NPS	24·86	CF241733	B3	MT791931
24	15/06/2018	34·17	F	URTI, headaches, fever	NPS	23·97	CF287798	D1	MT789751
25	24/06/2018	4·00	M	Asthma, respiratory distress, digestive signs	NPS	37·52	CF301306	B3	MT795861
26	30/08/2018	50·33	F	URTI, pneumopathy	NPS	30·63	CF408376	D1	MT789752
27	30/08/2018	69·42	F	COPD exacerbation, fever	NPS	23·53	CF408680	D1	MT789750
28	09/09/2018	0·17	M	URTI, irritability, fever	NPA	21·09	CF425016	D1	MT789749
29	10/09/2018	0·17	M	URTI, irritability, fever	NPA	23·75	CF425314	B3	MT789741

30	14/09/2018	0·92	M	URTI, bronchitis-bronchiolitis, respiratory distress, fever	NPS	26·38	CF434664	B3	MT789748
31	14/09/2018	3·50	M	Digestive signs	NPA	32·58	CF434673	D1	MT789753
32	24/09/2018	66·17	M	COPD exacerbation	NPS	31·62	CF451498	D1	MT789754
33	25/09/2018	57·40	M	URTI, headaches, digestive signs, fever	NPA	36·18	CF452824	D1	MT789758
34	08/10/2018	69·91	M	Muscle weakness, altered mental status, respiratory distress, cardiac signs	NPS	27·27	CF473248	B3	MT789739
35	11/10/2018	54·25	F	URTI	NPA	25·75	CF479745	B3	MT789743
36	12/10/2018	53·83	M	Respiratory distress, pneumopathy, altered mental status, fever	NPS	20·02	CF482253	B3	MT789747
37	15/10/2018	0·11	M	URTI, respiratory distress	NPA	40·70	CF485820	D1	MT795859
38	16/10/2018	2·33	F	URTI, respiratory distress	NPS	29·48	CF487798	B3	MT789740
39	18/10/2018	0·08	M	URTI, bronchitis-bronchiolitis, hypotonia	NPA	29·64	CF490734	D1	MT789755
40	19/10/2018	5·00	F	URTI, fever	NPS	ND	CF493730	B3	NA
41	30/10/2018	65·20	M	Respiratory distress, pneumopathy, sepsis, altered mental status, multivisceral failure, digestive signs	BAF	31·62	CF511948	D1	MT795860
42	23/11/2018	41·75	M	URTI, asthma, fever	NPS	NA	CF551739	B3	MT789746
43	04/12/2018	7·83	M	URTI	NPS	NA	CF569939	B3	MT789738
44	05/12/2018	33·67	F	URTI, digestive signs	NPS	NA	CF572239	B3	MT789742
45	22/12/2018	0·08	M	URTI, respiratory distress, bronchitis-bronchiolitis, hypotonia, fever	NPA	NA	CF601568	B3	MT789745
46	26/12/2018	0·13	M	URTI, digestive signs, fever	NPA	NA	CF606093	B3	MT789744

Underlined specimen numbers match with obtention of whole genome sequence. The VP1 sequence of the strain CF307029 is already deposited (accession number: LN681336) [8]. Sequences MT795867-868 are partial VP1. No specimen material was available for the strains 176070 (MT795862), 177016 (MT795865) and 182035 (MT795866). URTI=upper respiratory tract infection. COPD=chronic obstructive pulmonary disease. NPA= nasopharyngeal aspirate. NPS= nasopharyngeal swab. BAF= bronchoalveolar fluid. ND= not detected. UT= untyped. NA= not available
